# Fluoroquinolone-Resistant *Salmonella enterica*, *Campylobacter* spp., and *Arcobacter butzleri* from Local and Imported Poultry Meat in Kumasi, Ghana

**DOI:** 10.1089/fpd.2018.2562

**Published:** 2019-05-06

**Authors:** Denise Dekker, Daniel Eibach, Kennedy G. Boahen, Charity Wiafe Akenten, Yvonne Pfeifer, Andreas E. Zautner, Eva Mertens, Ralf Krumkamp, Anna Jaeger, Antje Flieger, Ellis Owusu-Dabo, Jürgen May

**Affiliations:** ^1^ Department Infectious Disease Epidemiology, Bernhard Nocht Institute for Tropical Medicine (BNITM), Hamburg, Germany.; ^2^ German Centre for Infection Research (DZIF), Hamburg-Borstel-Luebeck, Germany.; ^3^ Kumasi Centre for Collaborative Research in Tropical Medicine (KCCR), Kumasi, Ghana.; ^4^ Robert Koch Institute (RKI), FG13 Nosocomial Pathogens and Antibiotic Resistance, Wernigerode, Germany.; ^5^ Institute for Medical Microbiology, University Medical Center Göttingen (UMG), Göttingen, Germany.; ^6^ Robert Koch Institute (RKI), Division of Enteropathogenic Bacteria and Legionella, Wernigerode, Germany.

**Keywords:** poultry, sub-Saharan Africa, enteric bacteria, antibiotic resistance, mechanisms of resistance

## Abstract

*Salmonella* and *Campylobacter* are important gastroenteric pathogens. *Arcobacter butzleri* is an emerging enteric pathogen. Data on the frequencies of these poultry-associated pathogens on meat products sold in sub-Saharan Africa are scarce. This study aimed to analyze the frequency of *Salmonella*, *Campylobacter*, and *Arcobacter* antibiotic resistance and underlying mechanisms of resistance to fluoroquinolones in locally produced and imported poultry sold in urban Ghana. Chicken meat was collected and cultured on standard media. Bacterial strains were identified by biochemical methods and by mass spectrometry. Antibiotic susceptibility was tested by disk diffusion. Ciprofloxacin-resistant strains were assessed for molecular mechanisms of resistance. Among 200 samples, comprising 34% (*n* = 68) from the Ghanaian poultry industry and 66% (*n* = 132) from imports, 9% (*n* = 17) contained *Salmonella*, 11% (*n* = 22) *Campylobacter*, and 26.5% (*n* = 53) *A. butzleri*. Higher overall contamination frequencies were found in local meat. Most common *Salmonella* serovars identified were Kentucky (*n*/*N* = 5/16; 31%) and Poona (*n*/*N* = 4/16; 25%). *Campylobacter* were *C. coli* (*n*/*N* = 10/19; 53%) and *C. jejuni* (*n*/*N* = 9/19; 47%). Resistance to fluoroquinolones was high with 63% (*n* = 10), 75% (*n* = 15), and 52% (*n* = 25) in *Salmonella*, *Campylobacter*, and *Arcobacter*, respectively. A link between *Salmonella* Kentucky [sequence type (ST) 198] and a ciprofloxacin minimum inhibitory concentration of 16 μg/mL was found. *Salmonella* Poona-ST308 revealed transferable *qnrB2* fluoroquinolone resistance genes. Markedly high frequencies of resistant *Salmonella*, *Campylobacter*, and *Arcobacter* predominant in locally produced meat represent a probable transmission reservoir for human infections. These findings highlight the need for implementation of surveillance systems that focus on food hygiene, use of antibiotics in animal husbandry, and continuous monitoring of the quality of meat products from imports.

## Introduction

Globally, 1 in 10 child deaths during the first 5 years of life results from diarrheal disease, causing ∼800,000 fatalities worldwide annually, most occurring in sub-Saharan Africa (SSA) and South Asia (Kotloff *et al.*, 2017). *Campylobacter* and nontyphoidal *Salmonella* (NTS) are among the leading bacterial pathogens isolated from patients with diarrhea in both developed and developing countries (Krumkamp *et al.*, 2015). These pathogens are predominantly transmitted through food products, with poultry meat being identified as one of the major reservoirs (Geilhausen *et al.*, 1996). Another emerging swine-, poultry-, and waterfowl-associated pathogens are *Arcobacter* spp. (Corry and Atabay, 2001), especially *Arcobacter butzleri*, previously described as cause of gastroenteritis and invasive bloodstream infections in humans (Collado and Figueras, 2011). In particular, NTS have continuously been associated with foodborne cross-border outbreaks worldwide with a recent outbreak early in 2018 in eight U.S. states (https://www.cdc.gov/salmonella/outbreaks.html). The steadily increasing global trade of meat products between developing and industrialized countries bears the risk of spreading the animal-associated pathogens across different countries and continents. This threat is mainly a problem in countries without surveillance systems where pathogens can be imported or exported undetected. A high risk is posed to SSA regions, where data on the frequency of the bacteria in both locally produced and imported poultry are scarce and poorly monitored.

*Salmonella* and *Campylobacter* isolated from humans and other animals with reduced ciprofloxacin susceptibility and multidrug-resistance profiles have been rapidly emerging in recent years, with considerably high reported prevalences from Asia, Europe, and Africa (EFSA and ECDC, 2015; Pham *et al.*, 2015; Abdi *et al.*, 2017). Antibiotic use in animal rearing for growth promotion and for the prevention and treatment of infections has been identified as responsible for the increase of multidrug-resistant bacteria in meat products (Kluytmans *et al.*, 2013). Although in industrialized countries steps have been taken toward the control of the use of antimicrobials in food-producing animals, in SSA the use of antimicrobials remains largely unregulated (Maron *et al.*, 2013).

This study aimed to investigate and compare the frequency of *Salmonella enterica*, *Campylobacter* spp., and *Arcobacter* spp. in imported and locally produced poultry sold in urban Ghana. In addition, antimicrobial resistance in the mentioned bacteria is examined with special emphasis on genetic mechanisms of resistance to fluoroquinolones.

## Materials and Methods

### Study site and sample collection

In a cross-sectional study from May to December 2015, 200 chicken meat samples from local Ghanaian poultry (fresh meat) and from imports (frozen meat) were collected in Kumasi, the capital of the Ashanti region of Ghana. Sampling was based on availability. In total, 75 supermarkets were identified for sample collection of imported frozen meat, which was kept in freezers. Each supermarket was sampled once and the origin of the meat from imports was retrieved from packaging. If meat from different countries was available, one piece from each country was sampled per supermarket. Chickens originating from small local farms were purchased live from 19 open markets. Each merchant was visited on one occasion only. The chickens, which had been kept in cages, were slaughtered on purchase. For each sample (fresh and frozen), ∼15 g of leg, including skin, was collected and placed into a sterile homogenizing plastic bag and returned to the laboratory at 4–8°C in a cool box.

### Bacterial detection and identification

The meat was homogenized in the collection bag using a pestle and mortar. The sample was evenly distributed among the different enrichment broths for *Salmonella* (Selenite Broth; Oxoid, Basingstoke, UK), *Campylobacter* (Preston No 2; Oxoid), and *Arcobacter* (*Arcobacter* enrichment broth; Oxoid). The selenite broth was incubated with loose tops at 35–37°C for 18–24 h in normal atmosphere. Preston- and *Arcobacter* enrichment broths were incubated at 35–37°C for 18–24 h with loose tops under microaerophilic conditions (CampyGen sachets in a candle jar; Oxoid). The selenite broth was subsequently cultured on Xylose Lysine Deoxycholate agar (Oxoid) and incubated at 35–37°C for 18–24 h in normal atmosphere. *Salmonella* strains were identified by a latex agglutination test (Oxoid) and by the analytical profile index test (API 20E; bioMérieux, Marcy l'Etoile, France). Preston No. 2 broth was further cultured on selective Karmali agar (Oxoid), using a filter technique as described by Atabay *et al.* (2003). The plates were incubated at 35–37°C for 18–24 h under microaerophilic conditions. Negative cultures were incubated for another 4 d. For *Arcobacter*, Müller Hinton agar (Oxoid) with 5% sheep blood was used for culture of the broth using the filter technique as described for *Campylobacter*. Plates were incubated for 18–24 h at 30°C in microaerophilic conditions (CampyGen sachets in a candle jar; Oxoid) and if negative, for 4 more days. Preliminary confirmation of *Campylobacter* and *Arcobacter* was done by oxidase test and Gram staining. All bacterial strains were sent to Germany on dry ice where species identification was confirmed by mass spectrometry (MALDI-TOF MS; Bruker UK Limited, England). *Salmonella* serotyping was performed following the White–Kauffmann–Le Minor scheme (Grimont and Weill, 2007).

### Antibiotic susceptibility testing

Antibiotic susceptibility was tested using locally available antibiotics by the disk diffusion method and interpreted following the European Committee on Antimicrobial Susceptibility Testing (EUCAST) guidelines (http://www.eucast.org). Quality control of susceptibility testing was performed according to EUCAST (QC table v.5). *Salmonella* were tested for ampicillin, amoxicillin/clavulanic acid, tetracycline, chloramphenicol, cefotaxime, ceftazidime, trimethoprim/sulfamethoxazole, and ciprofloxacin. *Salmonella* isolates exhibiting resistance to ampicillin, trimethoprim/sulfamethoxazole, and chloramphenicol were defined as multidrug resistant (Eibach *et al.*, 2016). Ciprofloxacin minimum inhibitory concentrations (MICs) for *Salmonella* were determined by Etest (Oxoid) and interpreted as ciprofloxacin resistant with an MIC >0.06 μg/mL. *Salmonella* isolates were screened for extended-spectrum beta-lactamase production (ESBL) using cefotaxime and ceftazidime disks.

*Campylobacter* were tested for tetracycline, ciprofloxacin, and erythromycin. Resistance to all three classes of antibiotics was defined as multidrug resistant (Magiorakos *et al.*, 2012). *Arcobacter* isolates were tested for ciprofloxacin. To date, no specific breakpoints for defining resistance in *A. butzleri* are available. Thus, for interpretation, the absence of a zone of inhibition for ciprofloxacin was considered as an indicator for quinolone resistance.

### Molecular characterization of *Salmonella* isolates

*Salmonella*, *Campylobacter*, and *Arcobacter* DNA were extracted using the QIAamp DNA Mini Kit (Qiagen, Hilden, Germany) following the manufacturer's instructions. *Salmonella* exhibiting ciprofloxacin resistance were further screened for plasmid-located genes [*aac(6′)Ib(-cr)*, *qnrA*, *qnrB*, *qnrC*, *qnrD*, and *qnrS*], contributing to aminoglycoside and fluoroquinolone resistance (Pfeifer *et al.*, 2009; Pietsch *et al.*, 2017). Furthermore, DNA gyrase and/or DNA topoisomerase IV genes (*gyrA*, *gyrB*, *parC*, and *parE*) were screened for point mutations (Chau *et al.*, 2007). For all *Salmonella* isolates, sequence types (STs) were determined by multilocus sequence typing according to a previously published protocol (http://enterobase.warwick.ac.uk).

Ciprofloxacin-resistant *Campylobacter* were assessed for point mutations in the *gyrA* gene as described by Tang *et al.* (2017). After shipment to Germany, 48 of 53 *A. butzleri* isolates could be grown. All isolates were examined for point mutations in the quinolone resistance-determining region (QRDR) of the *gyrA* gene using the amplification and sequencing primers described by Abdelbaqi *et al.* (2007). *Campylobacter jejuni* ssp. *jejuni* NCTC 11168 and 81-176, *C. coli* RM 2228, and *Arcobacter butzleri* LMG 6620 served as reference strains.

### Statistical analysis

Dichotomous variables were described using frequencies and their proportion. Prevalence ratios (PRs) along with their 95% confidence intervals (CIs) were calculated to show associations between dichotomous variables. Missing values were excluded from the analysis; hence, in some calculations the denominator differs. All analyses were conducted using Stata Statistical Software 14 (StataCorp LP, College Station, TX).

## Results

### Meat sources

In total, 200 chicken meat samples, comprising 68 (34%) samples from the local Ghanaian poultry industry and 132 (66%) from imports, were collected within Kumasi. The Ghanaian meat was exclusively sold at open markets, whereas imported meat was primarily sold in supermarkets/cold stores. Only frozen meat from the Netherlands (*n* = 4; 11%) and the United States (*n* = 1; 3%) was also collected from open markets. The majority of imported meat products originated from the Netherlands (*n*/*N* = 38/132; 29%), the United States (*n*/*N* = 31/132; 23%), and Brazil (*n*/*N* = 31/132; 23%). Other countries included Belgium (*n*/*N* = 8/132; 6%), Germany (*n*/*N* = 3/132; 2%), Poland (*n*/*N* = 3/132, 2%), Ireland (*n*/*N* = 2/132, 2%), and Turkey (*n*/*N* = 1/132, 1%). Countries of origin were not traceable for 11% (*n*/*N* = 15/132) of the samples.

**FIG. 1.**
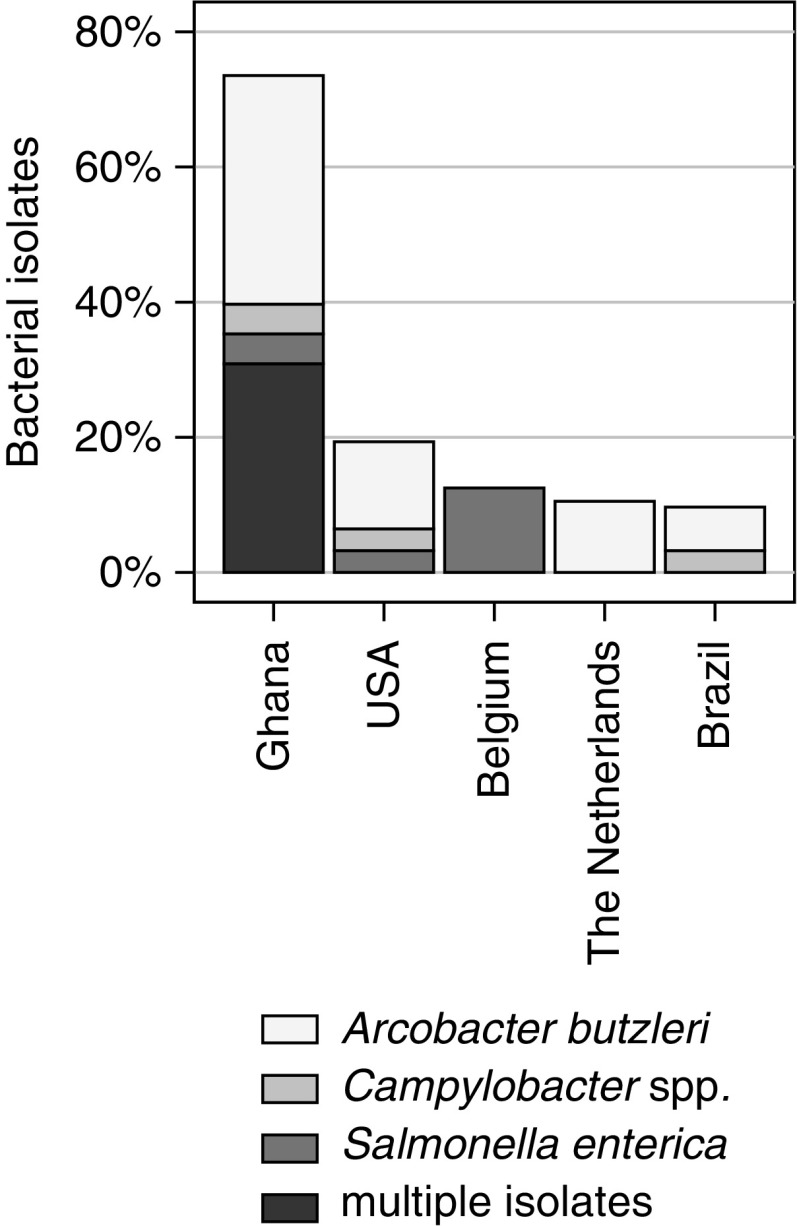
Frequencies of bacterial isolates in 200 meat samples of different origin. The category multiple isolates contains at least two different pathogens of *Salmonella enterica*, *Arcobacter butzleri*, or *Campylobacter* spp.

Isolates: of all collected samples, 9% (*n* = 17) were positive for *Salmonella*, 11% (*n* = 22) for *Campylobacter*, and 27% (*n* = 53) for *Arcobacter*. In local meat, *Salmonella* was detected in 19% (*n*/*N* = 13/68), *Campylobacter* in 28% (*n*/*N* = 19/68), and *Arcobacter* in 62% (*n*/*N* = 42/68) of the samples. In contrast, in imported meat, *Salmonella*, *Campylobacter*, and *Arcobacter* were found in 3% (*n*/*N* = 4/132), 2% (*n*/*N* = 3/132), and 8% (*n*/*N* = 11/132), respectively. Accordingly, there was a significant difference between locally sourced compared with imported meat products with higher frequencies in local meat for *Salmonella* (PR = 6.3; 95% CI: 2.1–16.6), *Campylobacter* (PR = 12.3; 95% CI: 3.8–40.1), and *Arcobacter* (PR = 7.4; 95% CI: 4.1–13.5). Multiple pathogens (pathogens under investigation found simultaneously) were primarily observed in local meat wherein 18 of 68 (26%) samples were concurrently positive with two different bacteria and 3 of 68 (4%) meat samples contained three bacterial genera. The country of origin of bacterial isolates from imported meat (considering countries with at least two samples) is summarized in Figure 1. *Salmonella* serovars identified included Kentucky (*n*/*N* = 5/16; 31%), Poona (*n*/*N* = 4/16; 25%), Agama (*n*/*N* = 3/16; 19%), Enteritidis (*n*/*N* = 2/16; 13%), Kaapstad (*n*/*N* = 1/16; 6%), and Ajiobo (*n*/*N* = 1/16; 6%). One serovar could not be identified due to loss of the isolate during culturing. *Campylobacter* comprised the species *C. coli* (*n*/*N* = 10/19, 53%) and *C. jejuni* (*n*/*N* = 9/19, 47%). Three *Campylobacter* spp. were lost during culturing, the species could not be identified. All *Arcobacter* spp. (*n*/*N* = 53/53, 100%) were *A. butzleri*.

Antibiotic resistance: the highest rate of resistance for *Salmonella* was revealed for tetracycline (*n*/*N* = 16/16; 100%) followed by ciprofloxacin (*n*/*N* = 10/16; 63%) and ampicillin (*n*/*N* = 9/16; 56%; Table 1). One imported *Salmonella* Agama isolate showed multidrug resistance. All *Salmonella* were sensitive to cefotaxime and ceftazidime with no isolates exhibiting ESBL production. The 10 ciprofloxacin-resistant isolates were all from Ghanaian poultry (except one sample whose origin could not be determined). Three ST198 *Salmonella* Kentucky isolates with a ciprofloxacin MIC of 16 μg/mL showed several mutations, including double mutations in *gyrA* and in *parC* (Table 2). Five isolates within the serovars Poona and Kentucky carried the plasmid-mediated quinolone resistance (PMQR) gene *qnrB2* and *qnrB19*, respectively. The four *Salmonella* Poona isolates carried additionally the aminoglycoside resistance gene *aacA4* gene.

**Table 1.** Antibiotic Susceptibility for Salmonella and Campylobacter

	*No. of resistant isolates (%)*	
*Drug*	Campylobacter *(*n* = 20)*	Salmonella *(*n* = 16)*
Ampicillin	—	9 (56)
Amoxicillin/clavulanic acid	—	5 (31)
Cefotaxime	—	0 (0)
Ceftazidime	—	0 (0)
Chloramphenicol	—	1 (6)
Ciprofloxacin	15 (75)	10 (63)
Erythromycin	6 (30)	—
Tetracycline	14 (70)	16 (100)
Trimethoprim/sulfamethoxazole	—	11 (69)

*Campylobacter*: two missing intermediate results interpreted as resistant; *Salmonella*: one missing; “—” indicates not tested.

**Table 2.** Mechanisms of Quinolone Resistance for *Salmonella*

*Serovar*	*Mechanism*	*MICcip (μg/mL)*	*No. of isolates*	*MLST*	*Country of origin*
*Salmonella* Enteritidis	D87G *gyrA*	0.25	1	ST11	Ghana
*Salmonella* Agama	D87G *gyrA*	2	1	ST28	Ghana
*Salmonella* Poona	T57S parC^**^, *qnrB2*	0.5	3	ST308	Ghana
*Salmonella* Poona	T57S parC^**^, *qnrB2*	0.5	1	Unknown	Unknown^*^
*Salmonella* Kentucky	D87Y + S83F *gyrA*; T57S, S80I *parC*	16	3	ST198	Ghana
*Salmonella* Kentucky	T57S *parC*, *qnrB19*	0.5	1	ST314	Ghana

^*^ Isolate imported: unknown country of origin.

^**^ T57S mutation in *parC* is not or doubtfully responsible for resistance phenotype (Wasyl et al. 2014).

CIP, ciprofloxacin; MLST, multilocus sequence typing; ST, sequence type.

Resistance in *Campylobacter* was highest for ciprofloxacin (*n*/*N* = 15/20, 75%) and tetracycline (*n*/*N* = 17/20, 85%). Multidrug resistance was detected in four strains (*n*/*N* = 4/20, 20%; Table 1).

As already mentioned, no breakpoints have been defined for *A. butzleri* susceptibility testing. However, 25 of 53 (47%) isolates were observed with no measurable zones of inhibition (ø < 6 mm) for ciprofloxacin. Although only single Thr86IIe *gyrA* mutations were detected in *C. coli*, two *C. jejuni* isolates revealed double or triple point mutations (Table 3). For *A. butzleri*, the Thr85Ile point mutation could be associated with quinolone resistance (Abdelbaqi *et al.*, 2007), which was detected in 15 isolates. These 15 isolates are a subset of the 25 isolates with no inhibition zone for ciprofloxacin (Table 3).

**Table 3.** Point Mutations in Quinolone Resistance-Determining Region of gyrA in Ciprofloxacin-Resistant *Campylobacter coli* and *Campylobacter jejuni* and *Arcobacter butzleri*

*Species*	*Mutation in* gyrA	*No. of isolates*
*Campylobacter jejuni*	Ser22Gly, Thr86Ile, Asn203Ser	3
	Thr86Ile, Asn203Ser^*^	1
	Thr86IIe	1
*Campylobacter coli*	Thr86IIe	9
*Arcobacter butzleri*	Thr86IIe	15

^*^ One isolate imported from Brazil; remaining isolates from Ghana.

## Discussion

Results from this study highlight the relevance of *S. enterica*, *C. coli*/*C. jejuni* and *A. butzleri* in poultry sold in Ghana. The proportion of contamination was higher for locally produced poultry than for imported meat products. In comparison, previous studies from Ghana found similar frequencies for *Campylobacter* and *Salmonella* in poultry, suggesting locally produced poultry as an important source for the spread of the studied bacteria (Sackey *et al.*, 2001; Adu-Gyamfi *et al.*, 2012). Numerous reports on poultry-associated NTS outbreaks due to contaminated meat products have been described worldwide, highlighting the potential of this pathogen to cause such outbreaks, which emphasizes the need for control especially within countries with weakened or absent surveillance systems (Niehaus *et al.*, 2011). Reports for *Arcobacter* are limited but the frequencies found in this study were lower than that found in a study conducted in Nigeria where 32% of chicken samples contained *Arcobacter* (Adesiji *et al.*, 2012). Furthermore, the demonstrated data showed that the microbiological quality with regard to the bacteria under investigation was better for frozen imported meat, which urges Ghanaian public health authorities and policy makers to enforce guidelines and regulations on food hygiene to meet the same hygienic standards. In the United States, a common practice is chlorination of meat products to reduce bacterial contamination (Paul *et al.*, 2017). Although chlorination has been banned in Europe (EFSA, 2010), a series of measures along the production line were implemented, such as protective clothing for farm workers, hygienic slaughtering practices, and adequate transportation and storage. In comparison, the freshly slaughtered animals at local markets in Ghana did not undergo any of the mentioned procedures.

As for frozen meat products, the increasing demand of chicken in Africa including Ghana has resulted in large amounts of imports from other countries. The United States, Brazil, and Europe (mainly the Netherlands and Belgium) are the main countries for poultry exports, having contributed to 66% of poultry imports to Ghana in 2016 (https://comtrade.un.org) and encompassing 82% among the samples from imports. Although low temperatures seem to have no significant effect on the viability of certain bacterial genera such as, for example, on *Escherichia* as outlined in a study by Eibach *et al.* (2018), *Campylobacter* spp. and *Arcobacter* spp. are known to show reduced viability when exposed to low temperatures such as freezing (Chan *et al.*, 2001). This effect could have been a further contributing factor accounting for lower frequencies in imported meat. Nevertheless, imported meat products do still represent a transmission reservoir for the studied bacteria. This observation was also highlighted by a European-wide baseline study conducted by the European Food Safety Authority (EFSA), which showed that member state prevalence in poultry varied from 4.9% to 100% for *Campylobacter* and 0% to 26.6% for *Salmonella* (EFSA, 2010). Country-specific differences were also seen in the EFSA conducted study, hence when looking at imported meat products, these should not be seen as a whole but data should be monitored country specific. As a rule, both imported and locally produced poultry could have been contaminated at all points of the animal production and food processing chain, including handling on the farm, during slaughter, and distribution. This fact should be taken into account when interpreting data. In either case, human contamination after product handling and consumption is unlikely but cannot be excluded. In contrast, cross-contamination from one animal to another during slaughtering could have occurred, which may have increased the overall number of bacterial isolates. However, regardless of the contaminating source, the studied bacteria found on sold meat products do represent without doubt a potential source of infections for the end consumer.

Antibiotic resistance to locally available drugs was considerably high. In particular fluoroquinolone resistance poses a significant public health challenge as it is among the key agents in use for the treatment of infections in Ghana. In animal husbandry, tetracyclines, penicillin/streptomycin combinations, and sulfonamides form the predominant antibiotic classes (Raufu *et al.*, 2014). For the same antibiotic classes, the highest resistance rates were observed in this study. There is strong evidence that antibiotic use in animal husbandry can lead to selection and then transmission of multidrug-resistant bacteria to humans (Chang *et al.*, 2015). Even though antibiotic treatment for infections that are typically self-limiting is not usually indicated, severe infections or invasive infections might require treatment. Hence, the spread of multidrug-resistant bacteria within communities, for which no alternative drugs are available, might compromise current treatment strategies in Ghana.

Quinolone resistance is typically a consequence of specific mutations in gyrase and topoisomerase IV genes (Aldred *et al.*, 2014). The present data demonstrate mutations in the QRDR of the *gyrA* gene of *S. enterica*, *C. jejuni*, *C. coli*, and *A. butzleri* isolates. Furthermore, previously reported PMQR genes and the aminoglycoside acetyltransferase gene *aacA4* have been detected in *S. enterica* isolates (Aldred *et al.*, 2014).

There was a significant contamination of Ghanaian poultry with the *Salmonella* serovar Kentucky (ST198). *Salmonella* Kentucky ST198 first emerged in Egypt and has since been associated with high-level ciprofloxacin resistance (Raufu *et al.*, 2014). In the past years, this ST has spread to several countries, including SSA, the Middle East, and Europe (Le Hello *et al.*, 2011). Previous investigations in broiler farms and slaughterhouses in Ghana demonstrated one dominant *Salmonella* Kentucky strain but the ST was not investigated (Andoh *et al.*, 2016). Another interesting ST found in this study was *Salmonella* Poona ST308 with plasmid-mediated resistance to ciprofloxacin. One isolate of this ST was recently reported from a human infection in Ghana (Kudirkiene *et al.*, 2018). Further studies are needed that focus on the spread and epidemiology of these circulating strains within Ghana. This study had a few limitations. Although for each bacterial detection ∼5 g of sample was used, larger quantities might have increased the level of bacterial detection. The sample size is quite small, was drawn only from urban areas, and focused on chicken originating from small farms. Therefore, our results may not be representative of all chicken consumed in Ghana.

## Conclusions

*Salmonella*, *Campylobacter*, and *Arcobacter* predominantly in locally produced meat represent a potential transmission reservoir for human infections. Also, the observed overall high level of resistance seen in locally available antibiotics, in particular, to fluoroquinolones is concerning. This level of resistance urges the implementation of strict surveillance systems that focus on food hygiene as well as on the use of antibiotics in animal farming. Furthermore, health authorities need to inform the public about emerging resistant strains in the communities such as the highly ciprofloxacin-resistant *Salmonella* Kentucky-ST198 and *Salmonella* Poona ST308; molecular investigations are the essential precondition for this. Surveillance programs also need to be coordinated with controlling the microbiological quality of imported meat and the food production end consumer chain by increasing awareness on food hygiene in the population.
